# Healingwith integrative management of Diabetic foot ulcer - A case report

**DOI:** 10.1016/j.jaim.2025.101174

**Published:** 2025-09-01

**Authors:** Rahul Katkar, K.T. Aadithyaraj, Usha Rana

**Affiliations:** a All India Institute of Medical Sciences, Rishikesh, Uttarakhand, 249203, India; b Dept. of Shalyatantra, All India Institute of Ayurveda, New Delhi, 110076, India; c Dept. of Shalyatantra, Uttarakhand Ayurveda University, Dehradun, Uttarakhand, 248001, India; d Dept. of Shalyatantra, Faculty of Ayurveda IMS Banaras Hindu University, Varanasi, UP, India

**Keywords:** Diabetic foot ulcer, Dushta vrana, Vrana upkrama, Jalaukavacharana

## Abstract

Diabetic foot ulcers (DFUs) occur in 15–25 % of diabetic patients at some point in their lifetime. The wide surgical debridement of the septic area is the cornerstone of diabetic foot ulcer treatment. There is a need to explore alternate therapies for DFU to reduce the risk of severe amputation. A 50-year-old male visited the Ayurveda OPD with a chronic ulcer on the plantar aspect of his right forefoot, which had persisted for two months. Ayurveda has considered *Dushta vrana* (∼septic nonhealing ulcer) and mentioned the *Vrana upkrama* (∼wound care measures) for its management. Among these *Vrana upkrama, Jalauka avcharana* (∼bloodletting using leech)*, Vrana Shodhana* (∼wound cleansing measures) by *Parisheka* (∼therapeutic streaming of medicated decoction)*, Vrana ropana* (∼wound-healing measures) are the three main wound care approaches along with *Shamana* (∼palliative therapy) for three months adopted in the present case for managing diabetic foot ulcers. DMIST tool was used to assess wound care at baseline and during follow-up. After three months of treatment, the integrated approach effectively shortens the healing time of DFUs, reduces the amputation rate and improves the standard of living of patients with DFUs with multiple Ayurvedic treatments.

## 1 Introduction

Diabetes mellitus is a chronic metabolic disease with a rising global prevalence. Diabetic foot ulcers (DFUs) are nonhealing wounds that often require extensive care. The primary cause of DFUs is diabetic neuropathy, with an annual incidence rate of 2 %–6 % among diabetic patients. Approximately 10 % of people with diabetes will experience a DFU during their lifetime [1]. Key risk factors include age over 50, a history of diabetes for more than ten years, uncontrolled blood sugar levels, peripheral neuropathy, peripheral vascular disease, and hypertension [2]. DFUs often lead to long-term medication use, extended hospital stays, and a higher risk of lower-limb amputation—10 to 30 times more common in diabetic patients. The healing process may take weeks to months, and recurrent infections frequently necessitate amputation [3]. The standard care for DFUs includes local wound care, surgical debridement, wound off-loading, vascular assessment, infection management, and glycemic control [4]. Ayurveda, through *Vrana upkrama* (∼wound care measures), provides complementary therapies for wound management [5]. Despite advanced surgical interventions such as debridement and infection control one major limitation of conventional DFU management is the high rate of recurrent infections. In cases where conventional treatments fail, amputation becomes the only option, permanently impairing the patient’s quality of life. This case suggests that Ayurvedic therapies can complement or even replace certain aspects of conventional care. The use of Ayurvedic polyherbal formulations suggests a comprehensive treatment plan that not only targets the wound but also systemic factors like inflammation and blood sugar regulation. This case study examines the efficacy of an integrative approach utilizing Ayurvedic practices to accelerate wound healing and reduce amputation risk.

## 2 Patient information

A 50-year-old male patient presented with a chronic ulcer on the plantar aspect of his right forefoot, which had been persisting for 2 months. He also reported difficulty in walking. Despite using antibiotics and hypoglycemic medicines for the past two months, he had no notable response and was advised for foot amputation. The patient had a history of uncontrolled diabetes and has been on medication for the past 10 years. The patient underwent 4th and 5th digit amputation of the right foot due to complications related to diabetes in a local hospital wound care center 7 years back. The patient had no history of hypertension, hyperlipidemia, coronary artery disease, smoking, and alcohol consumption. His hypoglycemic medication includes short, fast and long-acting insulin. The patient doesn't have any family history of diabetes mellitus.

## 3 Clinical findings

On local examination of the foot, the ulcer, measuring 8 × 6 cm (48 cm2), was situated on the plantar aspect of the right forefoot beneath the 4th and 5th metatarsal bones. The base of the ulcer is red, and the surrounding hyperkeratotic tissue is well-defined, undermining the skin margins. The floor of the ulcer is a slough with an edematous and sloping edge. There was discharge from the wound with a foul-smelling odour. He demonstrates palpable dorsalis pedis and posterior tibial pulse in both feet. The capillary filling time is less than 3 seconds. He lacks pain or temperature sensation in either foot. When the wound was examined with a sterile probe, no bone could be palpated. The pulse was 80/min, and blood pressure was 130/70 mm Hg. There was mild pallor, no icterus, no clubbing, no cyanosis. He also had a history of peripheral neuropathy, mainly affecting lower limbs. Cardiovascular examination revealed S1 and S2 audible and with no murmur. The rest of the physical and systemic examination was unremarkable.

## 4 Diagnostic assessment

All routine blood investigations were performed on admission and before discharge, including a complete blood count, glycosylated hemoglobin (HbA1c), fasting and postprandial blood sugar assessments mentioned in Table 1. HIV and hepatitis B surface antigens are non-reactive. After wound assessment, the wound is assigned a grade of 2 in Meggitt-Wagner's (1981) classification of the diabetic foot ulcer [6]. The diagnosis was made based on clinical findings as *Madhumehajanaya dushta vrana* (∼septic non-healing diabetic ulcer).

**Table 1:** Investigations.

Investigations	On admission (15/1/22)	Before discharge (16/3/2022)
Hemoglobin	8.3 gm/dl	9.1 gm/dl
RBCs	3.41	3.52
Wbcs	36.55	16
HbA1c	10 %	6.2 %
FBS	192.20 mg/dl	140.38 mg/dl
PPBS	263.9 mg/dl	178 mg/dl

## 5 Timeline of events

**Fig. 1:**
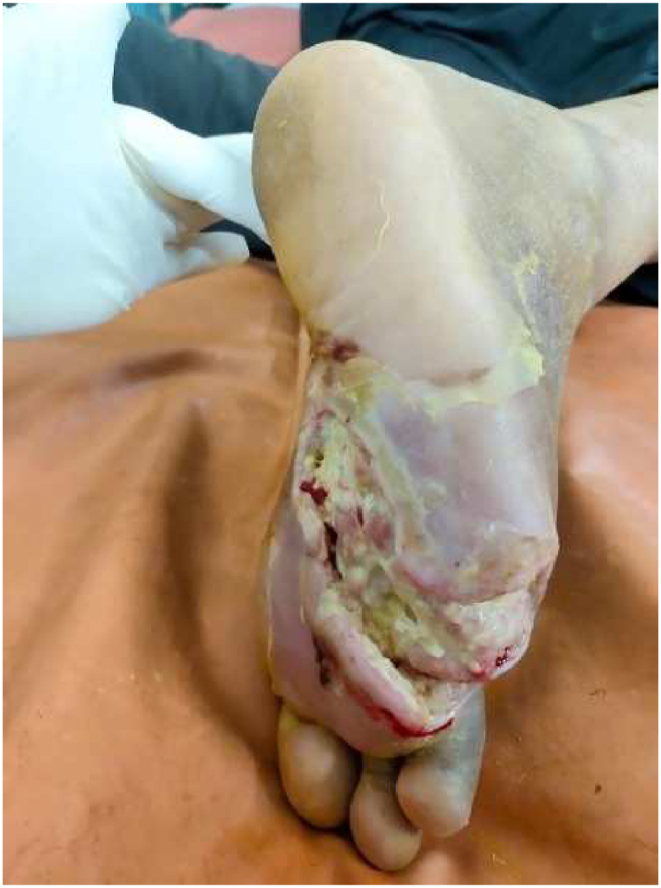
Wound at baseline. size ∼8 × 6 cm.

The case report's timeline is shown in [Table 2]. The patient was admitted to the *Shalya* IPD for 2 months, during which they were permitted to continue their hypoglycemic medications (insulin). (Fig. 1)

**Table 2:** Timeline.

Date	Observation	Intervention
15/01/22	The patient visited Ayurveda OPD & admitted to *Shalya* IPD	Clinical assessment & laboratory investigations were done
15/01/22 to 28/01/22	Ulcer to the plantar aspect of his right foot	Wound debridement + *Jalaukavacharana* done on alternate days for 2 weeks (7 sittings) + *Parisheka with Nyagrodhadi kashaya* (∼decoction) done daily and internal Ayurveda medicines started for 3 months.
29/01/22 to 28/02/22	Healthy granulation tissue started to appear	*Jalaukavacharana* was done weekly for up to 1 month (4 sittings) + *Parisheka* with *Nyagrodhadi kashaya* was done daily + dressing with *Jatyadi taila* (∼oil) was started and internal Ayurveda medicines continued.
1/03/2022 to 15/03/2022	Significant wound contraction with healthy granulation.	dressing with *Jatyadi taila* done daily and oral Ayurveda medicines continued.
16/03/2022	Partially healed wound	The Patient was discharged with advice to continue oral Ayurveda medicines.
16/04/2022	Completely healed wound	The Patient came for 1st follow-up. After assessment, the oral Ayurveda medicines stopped.
15/07/2022	Intact skin, no recurrence.	The Patient came for routine post intervention follow-up.

**Table 3:** Posology of drugs administered to the patient throughout the intervention period.

Sr.N	Drug	Route	Dose Regimen
1	Inj. Ceftriaxone + sulbactam	IV	1.5g (12hrly) for 7 days
2	Inj. Insulin	SC	Regular (5U– 5U– 4U) for 2 months Lantus 10U for 15 days
3	*Trayodashanga Guggulu* [10]	Oral	500 mg twice daily (post-meal with lukewarm water)
4	*Guggulutikta Kashaya* [11]	Oral	10 ml twice daily (at 6 a.m. & 6 p.m. empty stomach, with lukewarm water)
5	*Nisha-Amalaki Churna* [12]	Oral	5 gm once daily (before breakfast with lukewarm water)

## 6 Therapeutic intervention

**Fig. 2:**
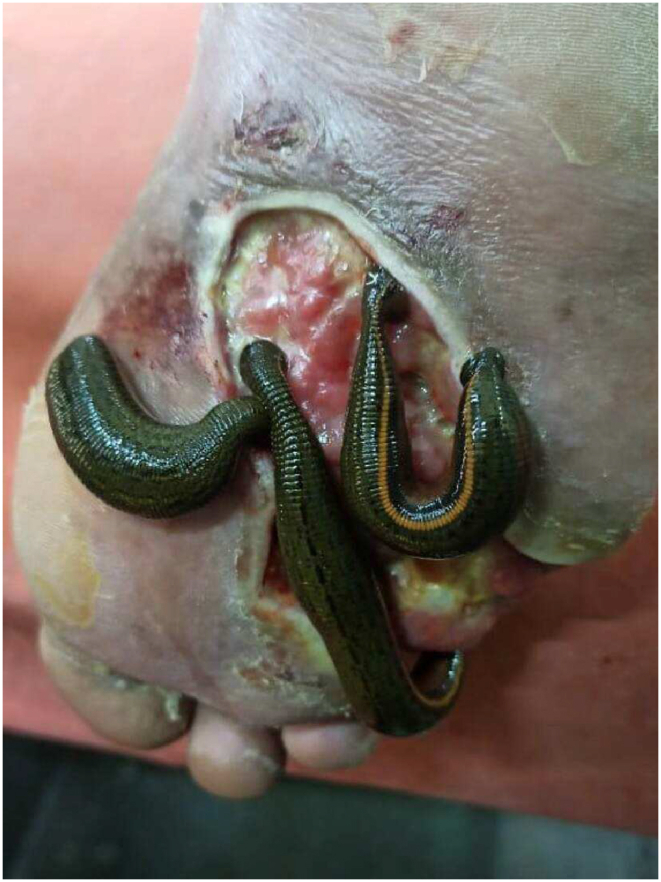
Wound on day 1. (Leech application) size ∼ 8 × 6 cm.

**Fig. 3:**
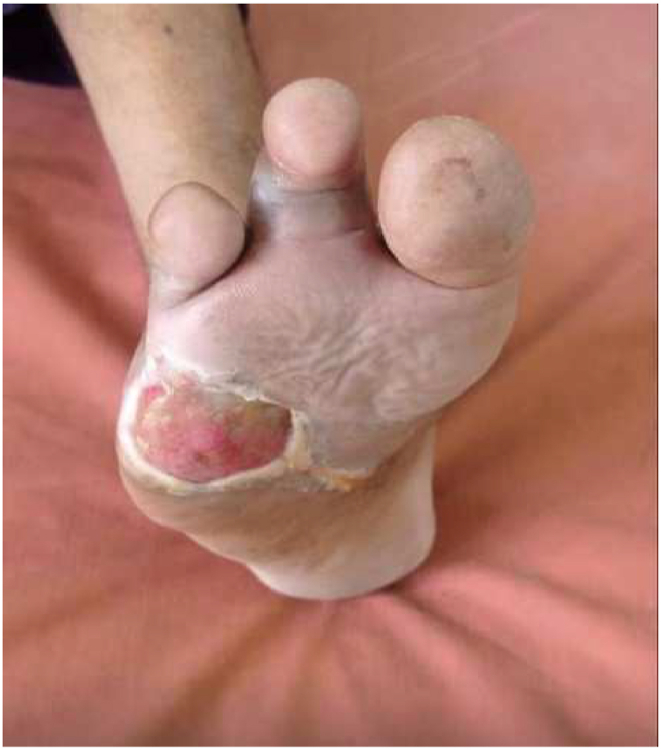
Wound on day 45. size ∼4 × 4 cm.

**Fig. 4:**
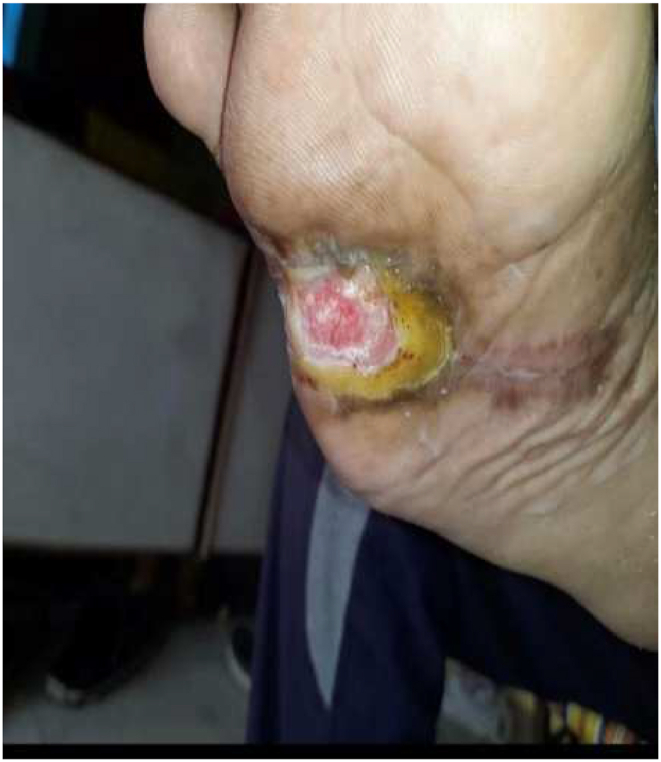
Wound on day 60. size ∼2 × 1 cm.

Following the primary assessment, wound culture was done. Then, prophylaxis using broad-spectrum antibiotics was started. The wound debridement was performed to eliminate remaining foreign particles and non-viable tissues (Fig. 2, Fig. 3, Fig. 4).

1. *Jalaukavacharana* [7] - The *Jalaukavacharana* which is done on alternate days for 2 weeks and then weekly for 1 month. A total 11 sittings of *Jalaukavacharana* were done in 45 days.
2. *Vrana Shodhana* by *Parisheka* - Following *Jalaukavacharana*, the wound bed was meticulously irrigated using a 50 ml syringe filled with *Nyagrodhadi kashaya.* [8] Subsequently, the dressing was performed using gauze soaked in *Nygrodhadi kashaya*, and bandaging was applied with a splint over the plantar surface daily for 60 days.
3. *Vrana ropana* - Following two weeks of consistent dressing with *Nyagrodhadi kashaya*, the wound bed exhibited healthy pink granulation tissue. Then, daily dressing of the wound with *Jatyadi taila* [9] was performed under aseptic precautions and was done on a regular basis for 75 days.
4. *Shamana* - Along with that, the patient was prescribed oral Ayurvedic medicine daily for 90 days from starting of the intervention. The posology of the drugs is shown in [Table 3].

## 7 Follow-up and outcome

Upon discharge, the patient exhibited partial wound healing and was instructed to return to the hospital for a follow-up appointment after one month. He was advised to continue taking oral medicines daily and to perform proper dressing with *Jatyadi taila* on the wound daily for the following month. After one month, the wound had completely healed. The patient was further instructed to return for a routine follow-up appointment in six months to assess for any signs of recurrence. Photographs of the wound were taken at baseline and at each follow-up visit. The DMIST (Diabetic foot ulcer healing scale) tool was used for wound assessment because it includes several parameters such as deep, maceration, infection, size, and tunneling, all of which are essential in wound care management and have a significant impact on wound healing (Table 4) [13].

**Table 4:** The scoring of diabetic foot ulcer with the Diabetic Foot Ulcer Healing Scale.

Objective symptoms	Before Treatment (15/01/22)	Interim analysis (01/03/2022)	At the time of discharge (16/03/2022)	At first follow-up (16/04/2022)	At post intervention follow-up (15/07/2022)
Depth^a^	2	1	1	0	0
Maceration†	2	0	0	0	0
Inflammation/Infection^b^	2	1	0	0	0
Size^c^	7	5	2	0	0
Tissue type of wound bed^d^	2	1	1	0	0
Type of wound edge^e^	2	1	1	0	0
Tunneling or undermining^f^	2	1	0	0	0
Total DMIST Score & Interpretation	14 Wound at baseline	10 Wound at interim analysis	5 Partially healed wound	0 Completely healed wound	0 Intact skin, no recurrence.

a Depth: 0-Intact, 1-Superficial layer/epidermis, 2-Subcutaneous/dermis to fatty tissue. †Maceration:0-None, 1- Slight only at wound edge.

b Inflammation/Infection: 0-None, 1-Signs of inflammation (e.g. warmth, erythema, swelling, pain), 2- Signs of local inflammation (eg. induration, pus, foul odour).

c Size: 0-Intact, 1- ⩽ 1 cm^2^, 2 - 1cm^2^ to⩽ 4 cm^2^, 3–4 cm^2^ to ⩽ 9 cm^2^, 4–9 cm^2^ to ⩽ 16 cm^2^, 5–16cm^2^ to ⩽ 25 cm^2^, 6- 25 cm^2^ to ⩽ 36 cm^2^, 7- 36 cm^2^ to ⩽ 49 cm^2^.

d Tissue type of wound bed: 0-Intact, 1- Granulation tissue, 2- white, yellow, and/or grey necrotic tissue.

e Type of wound edge: 0- Complete epithelialization, 1- too shallow to assess, 2- Hyperkeratosis.

f Tunneling or undermining: 0-None 1- ⩽ 2 cm, 2- 2 cm to⩽ 4 cm.

## 8 Discussion

Diabetic foot ulcers significantly impact patients’ quality of life, with standard medical treatments achieving only a 30 % healing rate within 20 weeks [14]. *Jalaukavacharana* (Leech Therapy), analogous to surgical debridement, aids in wound cleansing by removing necrotic tissue, reducing local inflammation, and enhancing microcirculation through anticoagulant properties. Similar studies confirm that leech therapy accelerates wound healing and prevents ischemic complications. This integrative approach aligns with evidence from other case series that report improved healing outcomes and lower amputation rates [15]. The *Nyagrodhadi Kashaya* herbal decoction serves a function similar to modern wound irrigation but offers additional antimicrobial and anti-inflammatory benefits. Literature supports its efficacy in reducing bacterial load and promoting granulation tissue formation, enhancing wound healing outcomes [16]. The *Jatyadi Taila* formulation promotes a moist healing environment and accelerates re-epithelialization. Clinical studies highlight its role in improving collagen fiber deposition, which was reflected in this case by complete epithelialization within three months [17].

The polyherbal formulations used in this study, including *Trayodashanga Guggulu* and *Guggulutiktaka Kashaya*, further contributed to the healing process. These formulations exhibit anti-inflammatory, antimicrobial, and immunomodulatory properties [18].^,^ [19] *Nisha Amalaki churna* combines two Ayurvedic herbs- *Haridra* (*Curcuma long Linn.*) and *Amalaki* (*Emblica officinalis. Garten*) in equal parts. *Haridra* and *Amalaki* are considered the best single drugs indicated for *Prameha* (∼Polyuria disorders) [20]. Other research supports their efficacy, with studies demonstrating their ability to inhibit inflammatory cytokines and promote tissue regeneration in chronic wounds. The observed reduction in HbA1c levels from 10 % to 6.2 % in this case aligns with other findings that show the potential of Ayurvedic formulations to assist in glycemic control, which is crucial for DFU management. This case study also reported no recurrence of the ulcer at six months of follow-up. This finding aligns with a case series where integrative approaches, including Ayurveda, resulted in better long-term outcomes and lower recurrence rates compared to conventional therapies alone. In DFU case series, Ayurvedic treatments focus on cleansing (*Vrana Shodhana*), healing (*Vrana ropana*) and promoting granulation tissue [21].

This study has several limitations. Subjective assessments, despite using the DMIST scale, may introduce observer bias. Incorporating objective wound assessment tools, such as digital imaging and biomarkers like IL-6 and VEGF, would provide a more comprehensive evaluation of healing progression [22]. Glycemic control variability due to the patient’s prolonged diabetes history posed an additional challenge. Improved monitoring and metabolic management would enhance outcome consistency. Furthermore, the individualized nature of Ayurvedic treatments may limit the generalizability of these findings.

This case reflects an acknowledgement of the need for more rigorous, large-scale studies to establish Ayurveda’s role in evidence-based DFU care. Future randomized controlled trials comparing integrative approaches with standard treatments are necessary to validate efficacy and establish standardized protocols. Long-term follow-up beyond six months is also needed to assess the durability of healing and recurrence risks. The sustained healing effect in this case may be attributed to the combined action of external therapies and internal medicines, which addressed both the local wound pathology and systemic metabolic dysregulation. The integrative approach resulted in faster healing, minimal scarring, and reduced risk of amputation.

## 9 Informed consent

The patient gave consent for his images and clinical information to be reported in a journal.

## 10 Conclusion

The standard care of diabetic foot management often involves multidisciplinary wound care, including wound cleansing and healing procedures. According to Ayurveda, these procedures align with *Vrana upakrama*, which includes *Jalaukavcharana, Vrana shodhana* and *Vrana ropana.* The integrated approach effectively shortens the healing time of DFUs, improves the clinical cure rate, reduces the amputation rate and improves patients' standard of living with multiple Ayurvedic treatments.

## Author contributions

RK: Methodology/Study design, Formal Analysis, Data Curation, Writing – Review & Editing, Visualization. AKT: Formal Analysis, Data Curation, Writing – Review & Editing, Visualization. UR: Conceptualization, Methodology/Study design, Formal Analysis, Investigation, Data Curation, Writing – Original Draft, Writing – Review & Editing, Visualization.

## Declaration of generative AI in scientific writing

None.

## Funding sources

None.

## Conflict of interest

The authors declare that they have no known competing financial interests or personal relationships that could have appeared to influence the work reported in this paper.
